# Microbial biocontrols in agriculture: An annotated selection of World Wide Web sites relevant to the topics in microbial biotechnology.

**DOI:** 10.1111/1751-7915.13569

**Published:** 2020-04-01

**Authors:** Lawrence P. Wackett

**Affiliations:** ^1^ Department of Biochemistry Molecular Biology & Biophysics, BioTechnology Institute University of Minnesota St. Paul MN 55108 USA

## Mechanisms of microbial control of plant diseases

https://www.frontiersin.org/articles/10.3389/fpls.2019.00845/full

This review article discusses how different microbes can convey protection against different plant infections via a range of very different mechanisms.

## Agbiome biocontrol products on the market

https://www.agbiomeinnovations.com/products/

Effective biocontrol products are currently on the market. This webpage put up by the company AgBiome highlights several of their microbial control agents. For example, “Howler” is composed of a *Pseudomonas chlororaphis* strain that controls pathogenic fungi in agricultural fields.

## Biocontrol products for farming and forestry

http://www.biocomes.eu/biological-control/

This website discusses biocontrol for sustainable farming and forestry. It is fairly basic but has useful videos and information explaining the basic elements of biocontrols.

## Novozymes biocontrol products

https://www.novozymes.com/en/advance-your-business/agriculture/crop-production/biocontrol

Novozymes is a large enzyme producer that also markets bacteri and fungi that target damaging pests that decrease crop yields in agriculture.

## Recommendations for promoting use of biocontrol agents

https://link.springer.com/article/10.1007%252Fs10526-017-9831-y

This journal article discusses different aspects of getting more biocontrol agents on the market. This includes more engagement with farmers and the public, and better mechanisms for going through regulatory approval.

## Fungi as biocontrol agents

http://www.angelfire.com/wizard/kimbrough/Textbook/FungiAsBiocontrolAgents_blue.htm

In addition to bacteria, numerous fungal strains are useful in controlling crops pests. This website contains many such examples.

## Emerging microbial biocontrol strategies

https://www.ncbi.nlm.nih.gov/pubmed/29362088

This review article discusses microbial biocontrol broadly, with some emphasis on microbiome aspects of microbial amendments onto plants and soils.

## Microbial biocontrols against soil‐born diseases

https://ec.europa.eu/eip/agriculture/sites/agri-eip/files/8_eip_sbd_mp_biocontrol_final.pdf

This page from eip‐agri discusses the economics, the microorganisms, and the potential impacts of microbial biocontrols.

## Bacillus thuringiensis

http://www.bt.ucsd.edu

*Bacillus thuringiensis* produces a parasporal protein crystal that is toxic to certain insects. This has been recognized for many decades and so *B. thuringiensis* is one of the first biocontrol agents to be widely used commercially. This website provides a good overview.

## Ecological aspects of biocontrol regulations

https://link.springer.com/article/10.1007/s10526-019-09964-y

This review article makes the point that biocontrol regulations in Europe generally treat biocontrol microbes as dangerous and prone to become dominant in the environment. Arguments against that view are provided.

## Biological control permits

https://www.aphis.usda.gov/aphis/ourfocus/planthealth/import-information/permits/regulated-organism-and-soil-permits/biological-control-organism-permits

This page discussed and provides documents for movement of biocontrol agents. It is a page of the United States Department of Agriculture.

## Genetically engineered microbial control agents

https://biotechnologie.rivm.nl/sites/default/files/2017-11/Future%2520introductions%2520of%2520genetically%2520modified%2520microbial%2520biocontrol%2520agents%2520in%2520the%2520EU.pdf

This report describes activities in genetic engineering of biocontrol agents and asks if current Europen Union legislation is currently adequate for proper risk assessment.

## Mosquito‐larvicidal toxin CryBa from *Bacillus thuringiensis* ssp. Israelenis

http://www.rcsb.org/structure/1W99

This site contains the deposited X‐ray structure and information on the protein toxic to the larae of *Aedes* and *Anopheles* mosquitoes.

## Rhizosphere bacteria protecting corn from fungi

https://springerplus.springeropen.com/articles/10.1186/s40064-016-1780-x

This article desribes the isolation and characterization of different bacteria that can protect corn from infectivity of *Fusarium verticillioides*.

## *Bacillus thuringienesis* review

https://microbewiki.kenyon.edu/index.php/Bacillus_thuringiensis

This Microbe Wiki pages covers *Bacills thuringiensis* ecology, metabolism, and utility in biocontrol.

## Biocontrols on grapevines

https://www.nature.com/articles/s41598-019-56741-z

This study compares biocontrols to more traditional copper fungicide for treating grapevine leaves susceptible to fungal infections.

## Comparative genomics of biocontrol *Bacillus* species

https://www.g3journal.org/content/10/3/881

This study examined the genomes of and possible virulence factors produced by different nematicidal *Bacillus* strains.

